# Navigating COVID-19 Surges: A Case Study of a Hospital-at-Home Implementation

**DOI:** 10.5334/ijic.8749

**Published:** 2024-12-19

**Authors:** Jin Wee Ng, Milawaty Nurjono, Michelle Mong Nee Kee, Hong Choon Oh, Qin Yong See

**Affiliations:** 1 Changi General Hospital, Singhealth, Singapore; 2 Centre for Population Health and Implementation Research, Singhealth, Singapore

**Keywords:** hospital-at-home, telehealth solutions, community-based care, early discharge strategy, remote clinical monitoring, COVID-19 pandemic response

## Abstract

This study documents the experience of implementing an adaptation of the Hospital-at-Home (HaH) model to alleviate the constraints in available hospital beds and manpower amid a surge in infection rates in Singapore during the Omicron and XBB COVID waves, addressing challenges and proposing insights for scalable implementation. HaH substitutes inpatient hospitalizations by leveraging existing community healthcare services and remote healthcare technologies. This HaH adaptation was designed to be activated in during surges and deactivated when bed and manpower demands stabilize, making it less intensive on hospital resources. HaH demonstrated success in facilitating safe early discharge and admission avoidance for high-risk patients, reducing hospital bed utilization without reducing care quality. However, challenges including lack of technological literacy, language barriers, and miscommunications resulting from clerical errors were experienced. Our findings suggest that hospitals with internal resource constraints can make adaptations to leverage existing providers and assets within the community where necessary. We also observed that HaH shifts many aspects of healthcare responsibility to patients and their caregivers, which may be beyond their expected capabilities. Clear communication of expectations and limitations from all parties involved is paramount to upholding the quality of care in HaH.

## Background

In recent years, public hospitals in Singapore have been experiencing a severe bed crunch, with patients spilling over to the Emergency Departments (ED) due to the increase in inflow and reduced outflow from an ageing population. (Paulo & Lim, 2023). This has drastic consequences; patients who require a hospital bed are instead being crammed into uncomfortable makeshift holding areas, waiting up to two days for a bed. This, in turn, restricts the ability of ambulances to release patients to the ED due to lack of space, hence, reducing the availability of ambulances [1,2]. In Singapore, it is widely recognized that building additional hospital wards is not a viable solution due to the land scarcity [3]. This had led to an increased focus on integrated care, which are health services that can be applied across various levels of care and coordinated both within and beyond the hospital [4], as a solution to alleviate the bed crunch. The Virtual Ward Model (VWM) is an integrated care approach that has demonstrated to be an effective solution to help healthcare systems cope with increasing demands for hospital resources. VWMs have been shown to significantly reduce unplanned hospital admissions without sacrificing patient safety, while having lower operating costs in some cases [5,6,7]. The VWM is typically made up of two components: (1) using predictive tools to identify patients who have a high risk of an unplanned readmission into inpatient care; and (2) reducing that risk by emulating the daily routines of a multi-disciplinary hospital ward in the community. While studies have shown that the VWM has had mixed results in the reducing unplanned readmissions, these studies also suggest that the VWM model has promising applications in significantly reducing bed utilization while maintaining the quality of patient care [8,9,10]. This has been shown to be particularly useful during surges of patient hospital admissions, particularly during a COVID wave [11,12]. In this paper, we describe our experience of implementing the Hospital-at-Home (HaH) VWM for the purposes of managing a COVID wave in Eastern part of Singapore and discuss its initial effectiveness in managing surges of patient inflow and providing care for patients.

### Virtual Ward Model: Hospital-at-Home

A government funded pilot programme was initiated by the Ministry of Health Office of Healthcare Transformation (MOHT) to bring the HaH model to mainstream due to the rising bed occupancy rates and patient overflow experienced in public hospitals [13,14]. HaH leverages contactless telehealth solutions. These solutions are primarily either remote clinical monitoring technologies (fall detection; vitals monitoring; portable investigations) or external tele-health service providers (24-hour teleconsultation and monitoring; integrated healthcare platform providers) among others [15]. HaH provides a viable substitute for inpatient hospitalizations through short-term home management, including treatments and monitoring of symptoms for durations ranging up to 2 weeks for patients with low to moderate acute conditions [16,17,18]. HaH also leverages existing services and infrastructure, such as patient homes and internet devices, and community resources like community nurses to facilitate for home visits tele-consultations, and remote clinical monitoring. This allows HaH to deliver daily care in a cost-effective way, enabling smoother transition to post-acute/post discharge care and allowing patients to benefit from hospital-level inpatient care while at their homes. On a larger scale, it aims to create an alternative channel for stable patients to be transferred to, increasing capacity within the ED for those who have urgent care needs [19,20].

### Outbreak of COVID-19 Omicron variants

**Figure 1:**
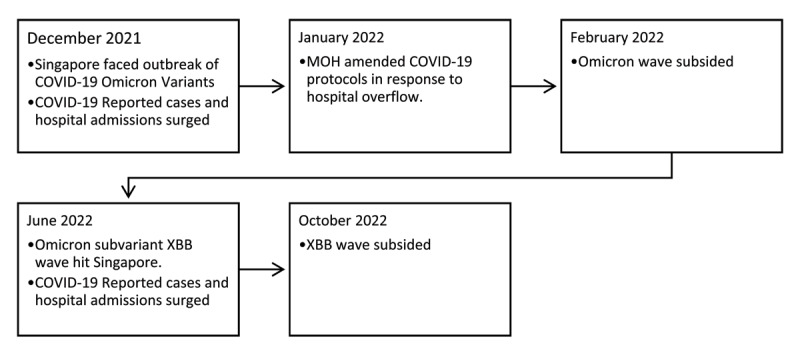
Timeline of Omicron and XBB Covid-19 Waves.

The Omicron variants of the COVID-19 virus emerged at the end of 2021. Compared to early COVID-19 strains, the Omicron variants spread at a higher rate [21,30], limiting the effectiveness of existing COVID vaccines developed for the earlier strains [22]. Singapore was severely affected by Omicron from December 2021 to January 2022 and the Omicron subvariant XBB from June 2022 to October 2022. Given the higher rate of transmissibility of the Omicron variant [23], the number of confirmed COVID-19 cases rose sharply, and hospitalization rates increased significantly. To mitigate overloading of hospitals due to the surge in the number of COVID cases, in January 2022, Singapore’s Ministry of Health [31], amended its COVID-19 healthcare protocols. In its revised protocol, processes were streamlined to focus on managing COVID-19 cases based on the severity of their symptoms and individual risk, with two Protocol levels for those who test positive for COVID-19 [24]. A timeline for these events is shown in Figure 1; Protocol levels are shown in Table 1.

**Table 1:** COVID-19 Health Protocol directives by Singapore’s Ministry of Health.


PROTOCOL LEVEL	INDIVIDUALS INVOLVED	SUGGESTED TREATMENT PLAN

1	High-risk (elderly; pregnant; paediatric; immunocompromised status etc.) Significant symptoms.	Assessed for placement in MOH Home Recovery Programme Management and monitoring in care facility.

2	Low risk, including children above 5 years old. Mild symptoms ascertained by primary care.	Consult primary care doctor. Avoid seeking hospital treatment for non-emergencies.


### Adaptation of HaH for COVID-19 Patients

Despite the streamlining of those affected by COVID-19 to their primary care doctors and HRP, hospital bed occupancy rates remained high. To ease the high bed occupancy arising from the surge, Changi General Hospital (CGH) commissioned a team made up of community nurses and physicians to implement an adapted version of the HaH care model to manage Protocol 1 patients who were not qualified for standard home recovery programme (HRP) yet required admission. This HaH adaptation served to facilitate safe early discharge and admission avoidance for COVID patients with higher medical complexity. This study aims to describe and assess the feasibility of HaH as an alternative care model.

## Method

### Patient Recruitment

Patients were selected based on a predefined selection criterion. Selected patients had to be clinically stable, had completed all major investigations and procedures, able to self-care or has a dedicated caregiver to carry out care needs, had access to telecommunication services (e.g. smartphone with internet connection), and stays within the service boundaries of the hospital (approximately 7 km radius from the hospital). Potential patients were identified either through referrals from inpatient wards or through a triage process conducted by the team, which involves the HaH team reviewing patient records in the hospital’s electronic database to identify potential patients based on the selection criterion. After these potential patients are identified, a secondary, in-person assessment was conducted by the clinician and nurses from the HaH team to further evaluate their condition and verify their eligibility. After the patient’s eligibility is confirmed, administrative personnel from the HaH team briefs the patient and their caregivers on the HaH program and provide them with any training or equipment necessary for the HaH treatment. Patients are then sent home via ambulance accompanied by a caregiver.

### Care Model and Implementation

The HaH team comprised of a small group of four staff, consisting of a physician, two nurses, and one operations staff specializing in continuity care, and managed the HaH service as an extension of their usual role. They were also supported by an external clinical “on-call” service available 24 hours a day, which provided acute care treatment and remote clinical monitoring for those with COVID-19 and required protocol 1 intervention at homes. These adaptations allowed the HaH care model to be adopted as a flexible ad-hoc service intended to be activated during a COVID-19 surge to deal with shortages of hospital beds and manpower, and subsequently de-activated once the COVID-19 wave subsided and manpower and bed shortages became more manageable.

This HaH service conducted investigations using biochemical and imaging studies, including full blood count, renal panel, COVID-19 Polymerase Chain Reaction (PCR) swab test and COVID-19 Antigen Rapid Test (ART). Medications were classified as symptomatic (e.g., paracetamol, cough syrups like dextromethorphan, antihistamines such as chlorpheniramine) or curative (e.g., antivirals like Paxlovid and IV Remdesivir). Patients enrolled into the program were required to measure their relevant vital signs (body temperature, blood pressure, blood oxygen, etc.) three times a day and transcribe their readings into an application that was installed into the patients’ smart device before their enrolment into the program, and the data was transmitted to the clinical team for review. The clinical team also conducted daily tele-consults and administered the necessary treatments via home-visits. To meet these operational requirements, the team worked extensively with external vendors. Partnerships included:

1. Partnership with an external care team from a local General Practitioner (GP) clinic to provide 24-Hour clinical-manpower support. This team consists of registered nurses and physicians and are equipped with the medications and consumables necessary. The primary and external teams reviewed their patients together during twice-daily handover meetings.
2. Partnership with a local technology company to facilitate the transmission of clinical monitoring data from the patient to the primary care team, and providing a dashboard for the primary team to view this data.
3. Partnership with ambulance and courier services to facilitate transporting patients and equipment. These services are used for non-emergencies and had an activation time of 2 to 3 hours.

The HaH service was activated during the Omicron and XBB COVID waves which caused a significant bed and manpower shortage. The HaH service enrolled 16 patients in March 2022 during the Omicron wave and 17 patients in October 2022 during the XBB wave. To assess HaH feasibility, we collected data on clinical outcomes, including adverse events during the HaH service period, length of stay, bed days saved, and 30-day readmission rates. Length of stay was calculated as the time between a patient’s transfer to HaH and discharge or transfer out. Bed days saved was calculated based on the sum of patients’ length of stay in HaH, and 30-day readmission rates was calculated based on the number of patient admissions to inpatient and emergency departments 30 days post-discharge from the HaH program. We also conducted a post-discharge phone call interview to collect feedback from patients enrolled into HaH. Table 2 provides a summary of the scope of this care model.

**Table 2:**
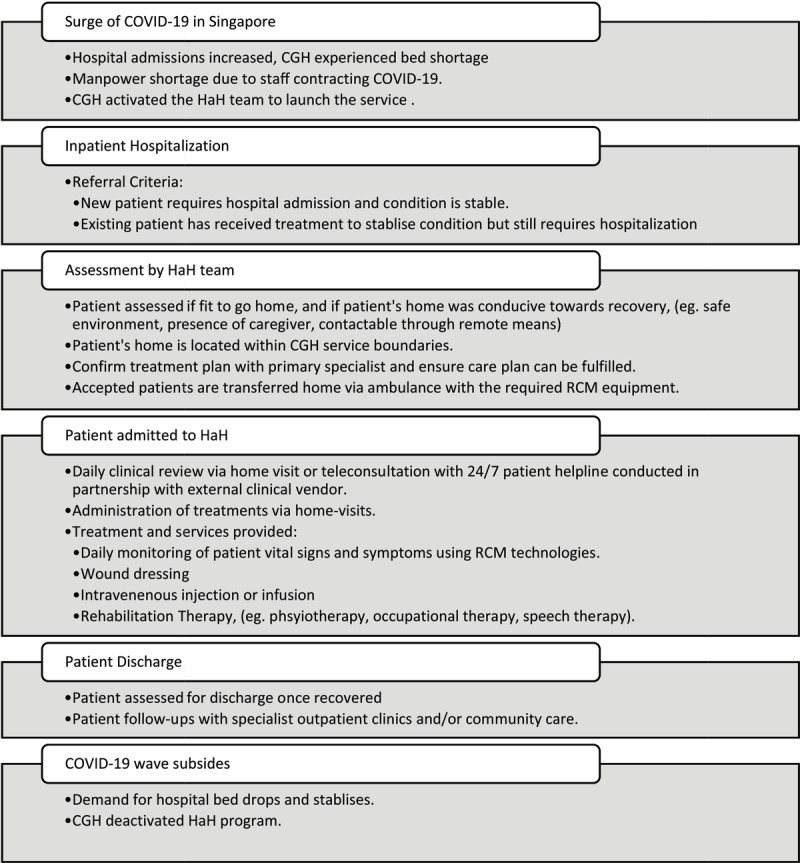
HaH Care Model and Patient Journey.

## Results

During the Omicron wave, HaH was activated for a total of 38 days. During the duration of the program, 16 patients (*Median age = 67*) were admitted into the HaH program, saving a total of 48 bed days. During the XBB wave, HaH was activated for 22 Days. During the duration of the program, HaH admitted 17 patients (*Median age = 73*) into the program, saving a total of 85 bed days. Throughout the duration of both programs, there were no adverse events reported (unexpected changes in medical condition; readmissions to hospital due to complications; adverse drug reactions). 30-Day readmission rates of discharged patients were zero. Patient demographics and full table of results are shown in Tables 3 and 4 respectively. No negative feedback was received during the post-discharge phone-call survey.

**Table 3:** Patient Demographics.


DEMOGRAPHIC	

Total number of patients	33

Median Age	72 (67, 73)

Gender Distribution	19 Males, 14 Females

Discipline of Referral Source	General Medicine (16),

	Geriatric Medicine (8),

	Respiratory Medicine (4),

	Infectious Diseases (3),

	Cardiology (2),

	Orthopaedic (1),


**Table 4:** HaH Descriptive Data.


METRIC	VALUE (OMICRON, XBB)

Program Duration (Days)	60 (38*, 22**)

Patients admitted	33 (16*, 17**)

Average length of stay	4 (3*, 5**)

Bed days saved	133 (48*, 85**)

No. of patients with unexpected changes in medical condition	0

No. of patients requiring readmission to hospital due to complications	0

No. of adverse drug reactions	0

30-Day readmission rate	0

Negative post-discharge feedback	0


*Value for Omicron Wave; **Value for XBB Wave.

## Discussion

The results are encouraging as the model of care has demonstrated unanimously positive outcomes. Echoing the literature on other VWMs, this application of HaH has demonstrated the potential of reducing hospital bed-utilization without compromising care quality and being relatively cost-efficient in terms of resources required [5,6,7]. We also showed that HaH can be successfully implemented with minimal allocation of hospital resources by leveraging on assets existing within the community and partnering with external providers. Unfortunately, this study’s low sample size limited our ability to provide reliable data and discuss certain markers in the HaH care model, such as reductions in unplanned readmission rates and operating costs demonstrated in other studies [5,8]. However, we will discuss the strengths, challenges and learning points that we have experienced in our implementation of HaH.

### Activation agility

Following the evidence in the literature, our results suggests that just like other VWMs, HaH is a viable solution to address surges in from COVID waves [11,12]. Furthermore, this study has shown that HaH was able to accomplish this as an adaptable service, saving on the extensive resources required to establish a permanent Virtual Ward. This adaptability was achieved by leveraging on existing structures and workflows within the healthcare system. An example of this approach is the engagement of nurses and physicians already embedded within the community to provide clinical support. By tapping into this pre-existing network, the program was able to optimize manpower and ensure a more cohesive and familiar healthcare experience for patients. Another example is the utilization of established discharge workflows to facilitate the transfer of patients and logistical processes to HaH. These examples expedited the implementation of the program but also minimized disruptions to patient care continuity, emphasizing the importance of aligning new initiatives with established protocols.

### Collaboration with community partners

VWMs like HaH can also take advantage of support from existing community care services. There are many community care support services already in existence within the residential areas of the patients enrolled to HaH. These support services are usually integrated within the community and have over many years built great rapport with their community and our patients, especially in residential areas with an ageing population. These support services are familiar with our patients and vice-versa, aiding our patients with daily tasks that they have difficulty with, sometimes acting as an alternative caregiver when a primary caregiver is not present. HaH require patients to take on self-responsibility and competency, as they must perform health monitoring and care activities independently and unsupervised by clinical staff. These support services empower patients to manage their new responsibilities, reducing reliance on clinical staff and improving manpower utilization.

### Challenges

#### Implementation

Due to manpower constraints, HaH was managed by a small four-person team and was limited in the number of concurrent patients the service could support. Furthermore, the existence of HaH as a referral path could not be properly communicated to the acute inpatient wards due to the urgency of its launch, leading to a slow start in referral numbers and consequently enrolments, which lead to the HaH team needing to personally triage patients. Referral numbers eventually increased as dissemination efforts and familiarity with the program improved, evidenced by the increased rate of enrolments during the second activation of the HaH service during the XBB wave.

#### Remote clinical monitoring

**Figure 2:**
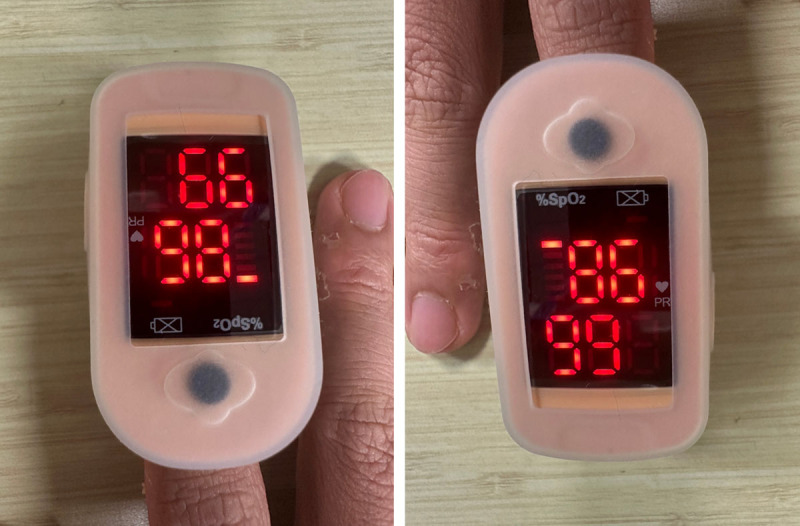
Transcription error due to orientation.

In the pursuit of optimal patient engagement of the RCM process within the framework of HaH, meticulous efforts were invested in pre-enrollment education for both patients and their caregivers. However, persistent challenges emerge, particularly among individuals with limited technological literacy. Issues encompass the operation of vital monitoring devices and the accurate transcription of readings into the digital database. Interestingly, some patients found that transcribing results to the digital dashboard via a smartphone app more challenging than operating the vital monitoring devices, which required just a single button press. Our experiences concurred with studies on RCM usage that found an increase in difficulty with an increase in the number of steps required to perform a task, particularly with the elderly [25]. Noteworthy issues also manifest in transcription errors, stemming from both human error and inexperience with RCM devices, as exemplified in Figure 2, where readings appear sensible from multiple device orientations, leading to misinterpretations when viewed from an unintended perspective.

A comprehensive approach to address these challenges involves targeted home visits for the purpose of re-educating patients on RCM procedures and receiving assistance from existing community systems. We provided an alternative in the form of regular nurse-led phone calls for patients experiencing difficulties with smartphone operation, in addition to home-visits from our nurses and community healthcare staff. While the support rendered by our medical staff was positively received, we acknowledge that this resulted in an increased workload, which has been also reported in other studies [26] prompting consideration for future enhancements. Transcription errors that lead to inaccurate readings reported to the clinical team were a major concern. False positives (abnormal readings for stable patients) waste manpower, while false negatives (normal readings for unstable patients) pose significant risks to patient safety, highlighting the need for technological advancements. Specialized clinical monitoring devices, particularly wearables that continuously track vitals and transmit readings to a digital dashboard, are a viable solution, as they have been shown to prevent errors, improve clinical team response times, and optimize manpower utilization [27,28,29]. This insight underscores the importance of considering such advancements in the planning and execution of future projects within the realm of remote clinical monitoring.

#### Language barriers

The operational nature of HaH shifts the responsibility of care to the patient and their caregivers through a holistic approach that prioritizes continuous training and open communication. We recognized that ongoing and transparent communication were essential to fostering a supportive and trusting relationship between the healthcare team, patients, and their caregivers. This constant exchange of information ensures that all parties involved are well-informed about the care plan, progress, and any adjustments needed. In the context of linguistic diversity, the program acknowledges the paramount importance of linguistic alignment within the primary care team. Therefore, the success of HaH is rooted in having a care team that comprehends and resonates with the linguistic diversity of the patient population.

#### External clinical vendor

In this HaH adaptation, our external clinical vendors helped to manage the care of our patients during periods where the primary HaH team was unable to support logistically with manpower. If the external vendor’s service scope is limited in certain aspects, it could inadvertently restrict the range of procedures or interventions that the primary HaH team initially intended to include. Therefore, the selection of an external clinical vendor becomes a crucial decision-making process. It was imperative that we chose a vendor whose capabilities align seamlessly with the scope of the HaH project. Beyond compatibility, it involves a thorough assessment of the vendor’s capacity to support and execute all the necessary procedures and interventions as outlined by the primary care team, which has ultimately contributed to the program’s effectiveness and the well-being of the patients it serves.

#### Awareness of patient’s abilities

Recognizing and accommodating the abilities of patients and caregivers is essential when assigning tasks or responsibilities. This awareness was underscored by an observation regarding our senior patients’ challenges with medications stored in childproof “push-and-twist” bottles. While seemingly minor, these issues can have significant implications, especially when time-sensitive medications are involved. Addressing such challenges is not just about rectifying isolated incidents; it pertains to a broader consideration for the scalability of projects. In the context of larger initiatives, the cumulative impact of addressing individual concerns, can result in a substantial manpower cost. This consideration becomes particularly crucial when envisioning the expansion or scaling up of projects. This challenge extends beyond medication administration; it applies to any procedure where the onus is placed solely on the patient or caregiver. We recommend that future projects adopt a proactive stance in anticipating and mitigating potential difficulties. By doing so, projects can enhance their efficiency, minimize unforeseen complications, and optimize resource allocation.

## Conclusion

An aging population places additional strain on the healthcare system, leading to heightened demands for hospital resources, extended hospital stays, and increased healthcare expenses. Our pilot investigation revealed that the implementation of HaH by CGH can effectively mitigates healthcare costs by facilitating early patient discharge, thereby substantially reducing the need for hospital bed days. This approach ensures that patients can recuperate in the familiar and supportive environment of their homes. The noteworthy success of our pilot study underscores the viability of this care model. We advocate for its widespread adoption across diverse demographic groups in the future, incorporating the refinements suggested in our study for continuous improvement and enhanced healthcare outcomes.
